# Phylogeography of Dengue Virus Serotype 4, Brazil, 2010–2011

**DOI:** 10.3201/eid1811.120217

**Published:** 2012-11

**Authors:** Marcio Roberto Teixeira Nunes, Nuno Rodrigues Faria, Helena Baldez Vasconcelos, Daniele Barbosa de Almeida Medeiros, Clayton Pereira Silva de Lima, Valéria Lima Carvalho, Eliana Vieira Pinto da Silva, Jedson Ferreira Cardoso, Edivaldo Costa Sousa, Keley Nascimento Barbosa Nunes, Sueli Guerreiro Rodrigues, Ana Barroso Abecasis, Marc A. Suchard, Philippe Lemey, Pedro Fernando da Costa Vasconcelos

**Affiliations:** Instituto Evandro Chagas, Ananindeua, Brazil (M.R.T. Nunes, H. Baldez Vasconcelos, D. Barbosa de Almeida Medeiros, C. Pereira Silva de Lima, V. Lima Carvalho, E. Vieira Pinto da Silva, J. Ferreira Cardoso, E. Costa Sousa Jr, K.N. Barbosa Nunes, S.G. Rodrigues, P.F. da Costa Vasconcelos);; KU Leuven, Leuven, Belgium (N. Rodrigues Faria, P. Lemey); Universidade Nova de Lisboa, Lisbon, Portugal (A. Barroso Abecasis);; University of California, Los Angeles, California, USA (M.A. Suchard);; and Universidade do Estado do Pará, Belém, Brazil (P.F. da Costa Vasconcelos)

**Keywords:** dengue virus, serotype 4, molecular epidemiology, phylogeography, Brazil, viruses, reemergence, genetic characterization, spatiotemporal patterns

## Abstract

Dengue virus serotype 4 (DENV-4) reemerged in Roraima State, Brazil, 28 years after it was last detected in the country in 1982. To study the origin and evolution of this reemergence, full-length sequences were obtained for 16 DENV-4 isolates from northern (Roraima, Amazonas, Pará States) and northeastern (Bahia State) Brazil during the 2010 and 2011 dengue virus seasons and for an isolate from the 1982 epidemic in Roraima. Spatiotemporal dynamics of DENV-4 introductions in Brazil were applied to envelope genes and full genomes by using Bayesian phylogeographic analyses. An introduction of genotype I into Brazil from Southeast Asia was confirmed, and full genome phylogeographic analyses revealed multiple introductions of DENV-4 genotype II in Brazil, providing evidence for >3 introductions of this genotype within the last decade: 2 from Venezuela to Roraima and 1 from Colombia to Amazonas. The phylogeographic analysis of full genome data has demonstrated the origins of DENV-4 throughout Brazil.

## Abstract 2

Multiple origins indicate this serotype was introduced in several episodes.

Dengue virus (DENV), a widespread arthropod-borne virus that commonly affects humans, belongs to the family *Flaviviridae*, genus *Flavivirus*, and is classified into 4 distinct serotypes (DENV 1–4). DENV is most prevalent in tropical and subtropical areas, where eco-epidemiologic conditions appear to sustain the virus. In particular, these regions harbor 2 competent DENV vectors (*Aedes aegypti* and *A. albopictus* mosquitoes) and have environments favorable for DENV (*1*). In past decades, the number of countries reporting DENV cases and those with endemic DENV has increased dramatically. These increases reflect the expanding habitat of the *Aedes* spp. mosquito vectors, the poorly planned urbanization of many cities in developing countries, an increased number of susceptible human hosts, and the rapid spread of DENV serotypes through global human travel networks (*2*–*4*). According to the World Health Organization, ≈3 billion persons living in >100 countries are at risk of being infected at least once annually by 1 of the 4 DENV serotypes (*4*).

In Brazil, DENV-1–3 were responsible for ≈5 million cases of DENV infection during 1990–2009, resulting in >15,000 reported cases of dengue hemorrhagic fever and ≈1,000 DENV-related deaths (*5*–*7*). DENV-4 reemerged in Brazil in 2010, 28 years after it was last detected in the country; the site of the reemergence was Roraima State, northern Brazil (*8*), the same state in which DENV-4 had last been detected in 1982 (*9*). Brazilian Ministry of Health data for 2010 and 2011 show there were 1,666,208 cases of DENV infection, including 26,659 severe cases and 1,097 associated deaths (*10*).

We describe the genetic characterization and spatiotemporal patterns of spread for DENV-4 strains isolated from 4 Brazilian states: Pará, Amazonas, and Roraima in northern Brazil and Bahia in northeastern Brazil. To characterize the origins of DENV-4 reemergence, we performed discrete Bayesian phylogeographic analysis on 98 full-length DENV-4 genomes and compared the results with those of a similar analysis on 314 envelope gene sequences.

## Material and Methods

### Viral Strains

We included 16 DENV-4 isolates in this study (Table). The viruses corresponded to low-passage virus strains (passage no. 1) from C6/36 cells obtained from the Department of Arbovirology and Hemorrhagic Fevers, Instituto Evandro Chagas, Brazilian Ministry of Health (Ananindeua, Brazil).

**Table:** Dengue virus type 4 strains isolated in Brazil used for genetic characterization and phylogeographic analyses

Identification	Strain	Year of isolation	Municipality, state, of isolation	GenBank accession no.
ROR 7542	Be H 774846	2010	Boa Vista, Roraima	JQ513333
ROR 7591	Be H 780090	2010	Boa Vista, Roraima	JQ513340
ROR 7620	Be H 780120	2010	Boa Vista, Roraima	JQ513341
ROR 7357	Be H 772846	2010	Boa Vista, Roraima	JQ513330
ROR 7363	Be H 772852	2010	Boa Vista, Roraima	JQ513331
ROR 7365	Be H 772854	2010	Boa Vista, Roraima	JN559741
STM 31	Be H 775222	2010	Santarém, Pará	JQ513334
BEL 83791	Be H 778494	2011	Belém, Pará	JQ513335
BEL 83846	Be H 778887	2011	Belém, Pará	JQ513337
BEL 83804	Be H 778504	2011	Belém, Pará	JQ513336
AM 5079	Be H 779652	2011	Manaus, Amazonas	JQ513339
AM 5105	Be H 780571	2011	Manaus, Amazonas	JQ513344
AM 4963	Be H 779228	2011	Manaus, Amazonas	JQ513338
AM 5090	Be H 780556	2011	Manaus, Amazonas	JQ513342
AM 5097	Be H 780563	2011	Manaus, Amazonas	JQ513343
BHI 3681	Be H 781363	2011	Salvador, Bahia	JQ513345

### Sequencing, Assembly, and Accession Numbers

Nearly complete genome sequences were obtained by using high-throughput sequencing on a GS FLX+ System (454 Life Sciences, Branford, CT, USA) as described (*11*). The sequences of the 5′ and 3′ termini were obtained by using 5′ and 3′ rapid amplification of cDNA ends systems (Invitrogen, Carlsbad, CA, USA), according to the manufacturer’s instructions, with the following specific sets of primers: DENV-4 5′SP1 (5′-AKCCCTGTCTTGGGTCCAGC-3′), DENV-4 5′SP2 (5′- TTGAACGCCTCTTGAAGGTC-3′), DENV-4 5′SP3 (CTCTTGAAGGTCCAGGTCTA), DENV-4 3′SP1 (5′-ATATCTGAATGGCAGCCATC-3′), and DENV-4 3′SP2 (5′-TCACTGGCTGTTTCTTCTGCT-3′). All 5′ and 3′ rapid amplification of cDNA ends amplicons were cloned into a plasmid bacterial system by using the TOPO TA Cloning Kit (Invitrogen) and directly sequenced (in both directions) by using the ABI Prism BigDye Terminator v1.1 Cycle Sequencing Kit on an ABI Prism 3130 DNA analyzer (Applied Biosystems, Foster City, CA, USA) with the M13F/M13R set of primers (Invitrogen).

Data generated by the GS FLX+ System and ABI 3130 platforms were assembled by using the mapping reference method as implemented by the GS Reference Mapper Program (available in Newbler v.2.6 software, http://454.com/products/analysis-software/index.asp). The mapper program was used to rearrange the reads against a given reference sequence by using the following default parameters: input = 20 bp, all contig threshold = 100, large contig threshold = 200, minimum overlap length = 40, minimum overlap identity = 70%, *k*-mer = 12 (seed step), and *k*-mer = 16 (seed length). A total of 16 new full-length DENV-4 sequences were obtained and deposited in GenBank (accession nos. JQ513330–JQ513345).

### Nucleotide Data Compilation and Alignment

We complemented our data with available full genome and envelope protein sequences for a total of 98 full genome (10,624 nt) and 314 full-length DENV-4 envelope protein (1,485 nt) sequences. These included 18 sequences from Brazil (3 from Belém, Pará State; 1 from Santarém, Pará State; 5 from Manaus, Amazonas State; 8 from Boa Vista, Roraima State; and 1 from Salvador, Bahia State); 26 full genome and 75 envelope protein sequences from the Caribbean region (Puerto Rico, Dominica, Dominican Republic, Jamaica, Puerto Rico, Trinidad and Tobago, Barbados, Montserrat, Martinique, and Bahamas); 8 full genome and 10 envelope protein sequences from Colombia; 34 full genome and 48 envelope protein sequences from Venezuela; 9 full genome and 88 envelope protein sequences from Mainland Southeast Asia (Thailand-Bangkok, Vietnam, Cambodia, Myanmar, and Peninsular Malaysia); and 3 full genome and 22 envelope protein sequences from Maritime Southeast Asia (Philippines, Indonesia, Singapore, and East Malaysia).

We aligned the sequences by using MAFFT (*12*). After we manually edited the resulting alignment by using Se-Al (*13*), the total lengths of the full genome and envelope protein alignments consisted of 10,624 bp and 1,485 bp, respectively. All sequences were screened for recombination by using the Phi-test (*14*), which is available in the SplitsTree4 program (*15*).

### Evolutionary Reconstruction of DENV-4 Dispersal

The parameters of a full probabilistic model of evolutionary history, including timed sequence evolution and spatial dispersal, were estimated by using a discrete Bayesian asymmetric diffusion approach (*16*) implemented in the BEAST software package (*13*,*17*). We used the Bayesian skyride model (*18*) as a flexible tree prior and a general time-reversible model with a discretized gamma distribution (general time-reversible + 4Γ) to account for among-site rate variation. To calibrate the time scale of the trees, we obtained isolation dates (in years) from the GenBank annotations, and to accommodate rate variation among lineages, we used a lognormal relaxed molecular clock approach (*19*).

This approach enabled us to estimate ancestral spatial locations throughout the phylogenetic history while accounting for uncertainty in the phylogenetic and diffusion process (*17*). For the full genome and envelope protein datasets, 3 Markov-chain Monte Carlo analyses were run for 50 million states and sampled once every 10,000 states. We used the BEAGLE library (*13*) together with BEAST (*13*) to augment the computational speed. After we removed 10% of the burn-in, we combined the runs by using LogCombiner (www.molecularevolution.org/software/phylogenetics/beast). Maximum clade credibility (MCC) trees were summarized by using TreeAnnotator and visualized by using FigTree (*12*). We use the SPREAD application (*20*) to visualize and convert the estimated divergence times and spatial estimates annotated in the MCC trees to a keyhole markup language file (the files are available from authors upon request). All evolutionary parameters are reported as posterior means along with their 95% Bayesian credibility intervals.

To obtain the expectations for the location state transitions, we estimated Markov jump counts (*18*,*21*) along the branches of the posterior tree distribution (*22*). We applied kernel density estimation in R to summarize Markov jump densities through time from particular locations.

## Results

### Asian DENV-4 Genotype I in Northern Brazil

The analysis (Phi-test implemented in SplitsTree) of the 98 DENV-4 full genomes did not provide support for recombination. Therefore, all 98 complete nucleotide (10,624 nt) and 314 (1,485 nt) envelope protein sequences were included in the Bayesian phylogeographic analyses by using a discrete asymmetric diffusion model and a flexible demographic prior (Technical Appendix Table).

**Figure 1:**
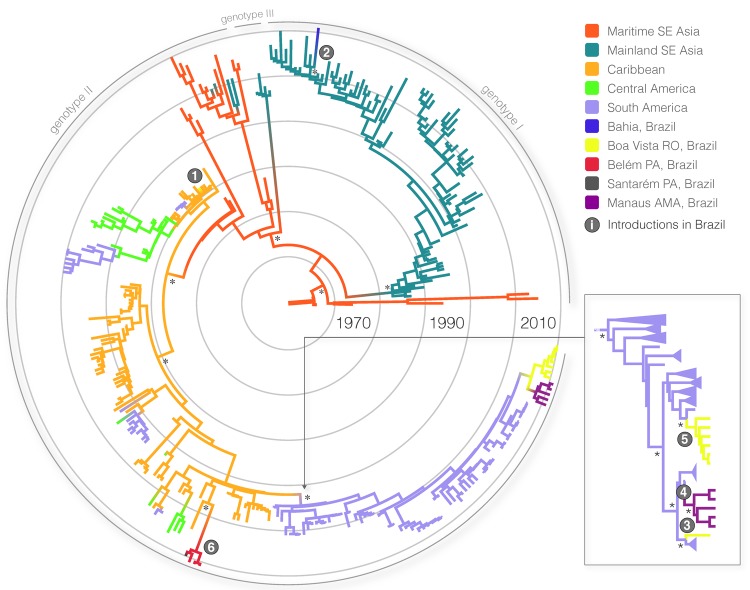
Maximum clade credibility tree demonstrating the phylogenetic relationships of the 314 dengue virus type 4 (DENV-4) envelope genes (1,485 nt). The major groups (genotypes I–III) are indicated. Branch lengths are scaled in time units; scale bars, representing the chronology of DENV-4 emergence, are color-coded according to the most probable geographic location of the descendent node. Introductions of DENV-4 in Brazil are indicated by numbered circles; numbering follows the temporal order of the mean estimate of divergence time for that particular sequence or clade. In selected nodes, * indicates posterior probability support of 1.00. Panel on right shows a detail of the maximum clade credibility tree (built by using an alignment of 98 DENV-4 full genomes [10,624 nt]) that reveals 3 distinct well-supported introductions of genotype II cluster from neighboring South American countries into Brazil. SE, Southeast; RO, Roraima State; PA, Pará State; AMA, Amazonas State.

The complete genome and envelope protein MCC genealogies confirmed that 3 major phylogenetic groups were compatible with the genotypes currently established for DENV-4: genotypes I–III (Figure 1). In particular, genotype I (n = 5 full genome, n = 85 envelope protein sequences) included strains that were isolated in Southeast Asia and a unique strain (BHI 3581 Be H 781363) that was isolated from an autochthonous febrile patient in 2011 in Salvador, Bahia State. The dense sampling in the envelope protein dataset made it possible for us to establish a relatively recent common ancestor with an Asian genotype I dating back to 2004 (95% Bayesian credible interval [BCI] 2002–2007). In particular, results from the genomic and envelope protein phylogeographic analyses confirmed with high posterior probability (>0.99) that the Brazilian strain from Bahia was introduced from Mainland Southeast Asia.

### Multiple Introductions of DENV-4 Genotype II in Brazil

Genotype II (n = 86 full genome, n = 223 envelope protein sequences) included the other 17 strains sampled in Brazil (Municipalities of Manaus, Belém, Santarém, and Boa Vista) and in Venezuela, Colombia, Central America, the Caribbean, and Southeast Asia. Genotype III (n = 2 full genome, 4 envelope protein sequences) included strains that were exclusively isolated from Southeast Asia (Thailand). On the basis of the envelope gene analysis, we reconstructed 3 introductions of genotype II DENV-4 in Brazil (Figure 1, left panel). First, the envelope protein MCC tree indicated a Brazilian cluster of strains identified in Boa Vista and Manaus as part of the major South American genotype II cluster. Second, the full genome MCC tree broke the cluster in Boa Vista and Manaus into 3 different lineages in the same genotype II cluster. Third, the large South American genotype II cluster, as part of the full genome MCC genealogy (Figure 1, right panel), indicates additional introductions of genotype II into Brazil. Thus, we identified a total of 6 introductions of DENV-4 in Brazil.

The most recent common ancestor of genotypes I–III is believed to have a Southeast Asian origin (7). In line with recent findings (*23*), we estimate that genotype II, the predominant genotype, was likely first introduced into the Americas (Caribbean region) around 1978 (95% BCI 1976–1979) (Figure 1; Technical Appendix Figure). Following a period of circulation in the Caribbean region, this genotype independently entered South American countries >4 times, as inferred respectively by the full genome and envelope protein analyses. Most of these introductions into South America seem to have led to relatively shallow phylogenetic clusters that probably reflect short-term survival of the virus in South America.

Nevertheless, our data provide support for a major genotype II cluster that reflects the successful establishment of this genotype in South American countries neighboring Brazil in or around 1994 (95% BCI 1992–1995). From Venezuela and Colombia, the genotype spread to Brazil >3 times in the last decade, as inferred by the full genome phylogeographic analyses (Figure 1, right panel). In contrast, the DENV-4 outbreak in Pará State (Santarém and Belém) was identified around 2008 (95% BCI 2006–2009) and is directly linked to strains from the Caribbean region. Overall, we found >3 distinct DENV-4 introductions into Roraima State: 1 from the Caribbean region ≈3 decades ago (indicated as 1 in Figure 1, left panel), and 2 from the strain that has become endemic in Venezuela within the last decade (indicated as 3 and 5 in Figure 1, right panel). One of these strains resulted in a well-supported cluster that dated back to about 2006 (95% BCI 2005–2007) and comprised 6 strains.

### Global Patterns of DENV-4 Dissemination

**Figure 2:**
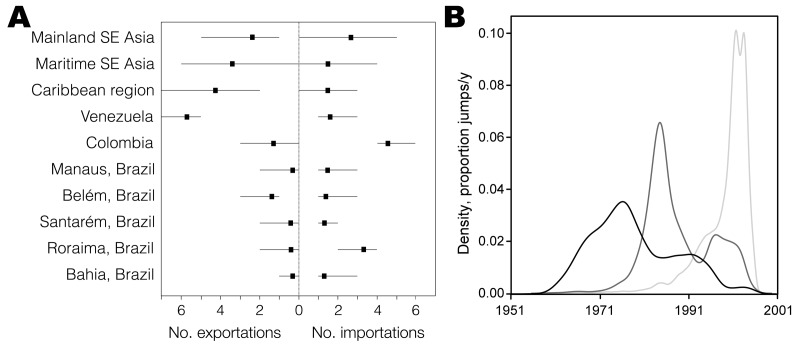
A) Estimated number of dengue virus type 4 exportations and importations (mean and 95% Bayesian credible intervals) (A). The full genome dataset was used and available data were discretized for countries neighboring Brazil. SE, Southeast. B) Markov jump density of viral exportations over time for the 3 major exporters of dengue virus type 4 (Maritime Southeast Asia, black lines; Caribbean region, dark gray lines; and Venezuela, light gray lines).

To identify the major export and import locations for DENV-4 at a global scale, we summarized the number of introductions from and to each of the locations that were estimated by using Markov jump count approaches as part of the full genome analysis (Figure 2, panel A). Our results pinpoint Maritime Southeast Asia, the Caribbean region, and Venezuela as the 3 most prominent sources of DENV-4 export. In addition, to investigate the temporal dynamics of viral dispersal from the 3 locations in the evolutionary history of DENV-4, we estimated the Markov jump density from these locations through time (Figure 2, panel B). We distinguished different peaks, which appear to reflect 3 distinct waves of DENV-4 migration; the most recent wave corresponded to exportations from South American countries bordering Brazil, particularly Venezuela, where DENV-4 has been established longer.

**Figure 3:**
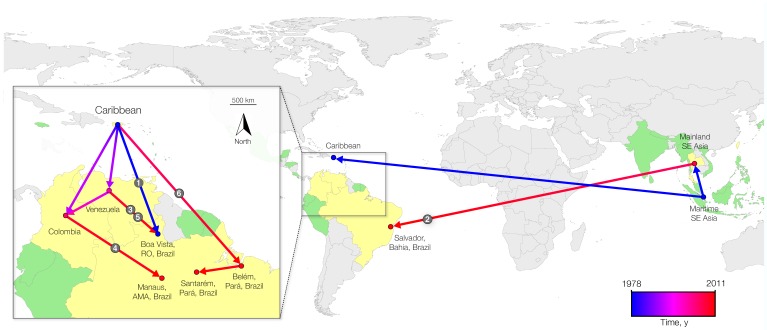
Overview of spatiotemporal dispersal of dengue virus type 4 (DENV-4) from Southeast (SE) Asia to the Caribbean region and then to South America. Links between geographic locations represent phylogeny branches of the full genome maximum clade credibility tree, as projected by using SPREAD software (*20*). The blue-red gradient is coded to the arrows and depicts the relative time that has elapsed since the earliest inferred viral migration out of Southeast Asia (i.e., 1978, 95% Bayesian credible interval 1977–1980). Introductions are numbered as in Figure 1. Green indicates the presence of DENV-4 where complete genomic sequence data were available; yellow indicates sampled countries where complete genomic sequence data were available; gray, indicates no genomic data available; and circles indicate sampled locations and are colored according to the earliest migration that was detected from the sink location. RO, Roraima State; AMA, Amazonas State.

After an initial establishment in the Caribbean region, genotype II most likely first dispersed to Brazil (Roraima State), Venezuela, Colombia, and, more recently, to Brazil (Belém, Pará State) again (Figure 3). Locations such as Santarém, Pará State, and Manaus, Amazonas State, do not have a direct relation to the Caribbean locations; instead, the strain found in Santarém is linked to a cluster of sequences from other locations in Pará State. This finding further indicates that there is ongoing circulation of DENV-4 within Brazil. Nevertheless, most recent introductions of genotype II in Brazil in the last decade seem to have been fueled by strains originating from bordering countries.

## Discussion

DENV-4 was not detected in Brazil for 28 years after the first clinical and laboratory reports of dengue fever cases in Roraima State during 1981–1982 (*9*). Since then, no additional cases were reported in the country until the reemergence of DENV-4 in Boa Vista, Roraima State, in 2010 (*8*). Following those first recent detections of DENV-4 in Brazil, the virus was identified in 2011 in the northern Brazilian states of Amazonas, Amapá, and Pará. Furthermore, DENV-4 was serologically detected in persons in several other states, demonstrating the potential for this virus to spread quickly to several regions (*8*).

Since the first detection of DENV-4 in Brazil in 1982 (*8*,*9*), partial genomic studies have confirmed that the original virus was directly associated with the Caribbean strains, which are known as genotype II. Genotype II has been responsible for several outbreaks in many countries in the Caribbean and South America (*8*,*24*–*28*). Phylogenetic analyses of different strains demonstrated the presence of 2 distinct genotypes (genotypes I and II) of DENV-4 in Brazil. The introduction of a new serotype and a distinct DENV-4 genotype (Asian genotype I) into Brazil after nearly 3 decades of no circulation highlights the potential for future outbreaks of genotype I in this country. The presence of a serotype against which the population is not immunized indicates that the country is at risk for a sharp increase in the number of dengue virus infections, including severe cases (*8*).

The phylogeographic analyses of full genomes and envelope genes confirmed the co-circulation of 2 distinct DENV-4 genotypes (I and II) in Brazil. Genotype II is most common in South America and the Caribbean region, and genotype I was represented by 1 strain isolated from Bahia State in northeastern Brazil. The analyses also confirmed the introduction of DENV-4 into Pará State from the Caribbean region and suggest that this introduction is very recent (Technical Appendix Figure).

Previous phylogenetic analyses have been performed on partial and a few complete DENV-4 genomes (*8*,*29*–*31*). The results we obtained by using the new set of complete genomes suggest that DENV-4 genotype II emerged and reemerged in Brazil from >3 distinct origins (Southeast Asia, the Caribbean region, and Venezuela), which demonstrates a dispersal pattern in Brazil that is far more complex than expected from standard epidemiologic data.

We demonstrate that full genome analysis complements the analysis of more widely available envelope protein sequences. Although a dense sampling may have its own particular benefits for divergence time and spatial diffusion estimation, full genomes can provide more phylogenetic resolution to unravel individual introductions from populations of closely related strains.

Our results indicate that DENV-4 was exported multiple times from the Caribbean region to northern South American locations before the virus became established in South America. Overall, the spatiotemporal patterns of DENV-4 revealed in South America over a long time scale are reminiscent of a source-sink model of virus dispersal (*32*), in which the location of primary source virus populations shifted from Maritime Southeast Asia to the Caribbean region and, most recently, to northern South America, albeit with co-circulation in each location. This dispersal pattern differs from the source-sink dynamics of seasonal influenza, in which each epidemic wave seems to be caused by emergence from the same source (*33*). Active molecular epidemiologic surveillance will be essential for better characterizing local source populations of DENV-4.

In conclusion, we found that 2 distinct genotypes (I and II) of DENV-4 are circulating in Brazil, and we provide insight into the origin and dispersal of the DENV throughout northern Brazil and areas of several South American countries. Further studies are needed to analyze complete genomes from other countries to which DENV-4 is endemic; such studies will more fully elucidate the geographic dispersal dynamics of DENV-4 in regions of the Americas where it is endemic.

Technical Appendix Dengue virus-4 strains used for phylogeographic analyses and Comparison of the divergence time of dengue virus 4 in Maritime Southeast Asia, the Caribbean, and Roraima, Pará, and Manaus States (Brazil), Brazil, 2010–2011.
