# Supplementary material for: Phylogeography of Dengue Virus Serotype 4, Brazil, 2010–2011
Source: Emerg Infect Dis. 2012 Nov;18(11):1858–64. doi: 10.3201/eid1811.120217 (PMC3559147; doi:10.3201/eid1811.120217)
Supplement: Technical Appendix — Dengue virus-4 strains used for phylogeographic analyses and Comparison of the divergence time of dengue virus 4 in Maritime Southeast Asia, the Caribbean, and Roraima, Pará, and Manaus States (Brazil), Brazil, 2010–2011. [file 12-0217-Techapp-s1.pdf]

Article DOI: http://dx.doi.org/10.3201/eid1811.120217

Phylogeography of Dengue Virus Serotype 4, Brazil, 2010–2011

Technical Appendix

Technical Appendix Table. Dengue virus-4 strains used for phylogeographic analyses according to genotype, location of isolation, date of isolation, and GenBank accession number, Brazil, 2010–2011

Strain	Genotype	Location	Date	GenBank accession no.
BHI3581 BeH781363	I	Bahia, Brazil	03/18/2011	JQ513345
ThD4_0087_77	I	Bangkok, Thailand	1977	AY618991
ThD4_0348_91	I	Bangkok, Thailand	1991	AY618990
ThD4_0485_01	I	Bangkok, Thailand	2001	AY618992
BID_V2166	II	Aragua, Venezuela	1998	FJ639739
BID_V2165	II	Aragua, Venezuela	1998	FJ639738
BID_V2164	II	Aragua, Venezuela	1998	FJ639737
BID_V2163	II	Aragua, Venezuela	1998	FJ639736
BID_V2173	II	Aragua, Venezuela	1999	FJ639745
BID_V2172	II	Aragua, Venezuela	1999	FJ639744
BID_V2170	II	Aragua, Venezuela	1999	FJ639742
BID_V2176	II	Aragua, Venezuela	2000	FJ850095
BID_V2177	II	Aragua, Venezuela	2000	FJ639748
BID_V2206	II	Aragua, Venezuela	2001	FJ639773
BID_V2194	II	Aragua, Venezuela	2001	FJ639764
BID_V1156	II	Aragua, Venezuela	2007	GQ868645
BID_V1155	II	Aragua, Venezuela	2007	GQ868644
BID_V1154	II	Aragua, Venezuela	2007	GQ868643
BID_V1153	II	Aragua, Venezuela	2007	GQ868642
BID_V1160	II	Aragua, Venezuela	2007	FJ182017
BID_V1158	II	Aragua, Venezuela	2007	FJ182016
BID_V1161	II	Aragua, Venezuela	2007	EU854301
BID_V1159	II	Aragua, Venezuela	2007	EU854300
BID_V1157	II	Aragua, Venezuela	2007	EU854299
BID_V2500	II	Aragua, Venezuela	2007	FJ882591
BID_V2499	II	Aragua, Venezuela	2007	FJ882590
BID_V2498	II	Aragua, Venezuela	2007	FJ882589
BID_V2497	II	Aragua, Venezuela	2007	FJ882588
BID_V2496	II	Aragua, Venezuela	2007	FJ882587
BID_V2495	II	Aragua, Venezuela	2007	FJ882586
BID_V2494	II	Aragua, Venezuela	2007	FJ882585
BID_V2493	II	Aragua, Venezuela	2007	FJ882584
BID_V2492	II	Aragua, Venezuela	2007	FJ882583
BID_V2491	II	Aragua, Venezuela	2007	FJ882582
BID_V2490	II	Aragua, Venezuela	2007	FJ882581
BID_V2489	II	Aragua, Venezuela	2007	FJ882580
BID_V2501	II	Aragua, Venezuela	2008	FJ882592
ThD4_0734_00	II	Bangkok, Thailand	2000	AY618993
BEL83804 BeH778504	II	Belém, Brazil	01/11/2011	JQ513336
BEL83791 BeH778494	II	Belém, Brazil	01/12/2011	JQ513335
BEL83846 BeH778887	II	Belém, Brazil	01/20/2011	JQ513337
ROR7357 BeH772846	II	Boa Vista, Brazil	07/17/2010	JQ513330
ROR7363 BeH772852	II	Boa Vista, Brazil	07/18/2010	JQ513331
ROR7465 BeH773583	II	Boa Vista, Brazil	08/20/2010	JQ513332
ROR7365 BeH772854	II	Boa Vista, Brazil	07/21/2010	JN559741
ROR7620 BeH780120	II	Boa Vista, Brazil	11/21/2010	JQ513341
ROR7542 BeH774846	II	Boa Vista, Brazil	08/24/2010	JQ513333
ROR 82 BeH402276	II	Boa Vista, Brazil	03/26/1982	JN559740
ROR7591 BeH780090	II	Boa Vista, Brazil	11/29/2010	JQ513340
12659201	II	Dominica	1981	AF326573
AM5105 BeH780571	II	Manaus, Brazil	01/13/2011	JQ513344
AM4963 BeH779228	II	Manaus, Brazil	01/14/2011	JQ513338
AM5079 BeH779652	II	Manaus, Brazil	01/24/2011	JQ513339

Strain	Genotype	Location	Date	GenBank accession no.
AM5090 BeH780556	II	Manaus, Brazil	01/29/2011	JQ513342
AM5097 BeH780563	II	Manaus, Brazil	01/29/2011	JQ513343
BID_V2610	II	Mérida, Venezuela	2007	GQ199876
BID_V3412	II	Norte Santander, Colombia	2005	GQ868585
H241	II	Philippines	1956	AY947539
BID_V1083	II	Puerto Rico	1986	EU854295
BID_V2430	II	Puerto Rico	1994	GQ199879
BID_V2429	II	Puerto Rico	1994	GQ199878
BID_V860	II	Puerto Rico	1994	FJ226067
BID_V2432	II	Puerto Rico	1995	GQ252675
BID_V2431	II	Puerto Rico	1995	GQ199880
BID_V2434	II	Puerto Rico	1995	FJ850057
BID_V2433	II	Puerto Rico	1995	FJ810417
BID_V2439	II	Puerto Rico	1996	GQ199885
BID_V2438	II	Puerto Rico	1996	GQ199884
BID_V2437	II	Puerto Rico	1996	GQ199883
BID_V2436	II	Puerto Rico	1996	GQ199882
BID_V2435	II	Puerto Rico	1996	GQ199881
BID_V2440	II	Puerto Rico	1996	FJ850058
BID_V1082	II	Puerto Rico	1998	FJ024424
BID_V1094	II	Puerto Rico	1998	EU854297
BID_V1093	II	Puerto Rico	1998	EU854296
BID_V2445	II	Puerto Rico	1998	FJ882598
BID_V2444	II	Puerto Rico	1998	FJ882597
BID_V2442	II	Puerto Rico	1998	FJ882596
BID_V2441	II	Puerto Rico	1998	FJ882595
BID_V2443	II	Puerto Rico	1998	FJ850059
BID_V2448	II	Puerto Rico	1999	FJ882601
BID_V2447	II	Puerto Rico	1999	FJ882600
BID_V2446	II	Puerto Rico	1999	FJ882599
BID_V1600	II	Santander, Colombia	1997	FJ024476
BID_V3409	II	Santander, Colombia	2001	GQ868582
BID_V3408	II	Santander, Colombia	2001	GQ868581
BID_V3407	II	Santander, Colombia	2001	GQ868580
BID_V3406	II	Santander, Colombia	2001	GQ868579
BID_V3411	II	Santander, Colombia	2004	GQ868584
BID_V3410	II	Santander, Colombia	2004	GQ868583
STM31 H775222	II	Santarém, Brazil	11/10/2010	JQ513334
Singapore_8976	II	Singapore	1995	AY762085
06K2270DK1	II	Singapore	2005	GQ398256
Taiwan_2K0713	II	Taiwan	2004	AY776330
ThD4_0017_97	III	Bangkok, Thailand	1997	AY618989
ThD4_0476_97	III	Bangkok, Thailand	1997	AY618988

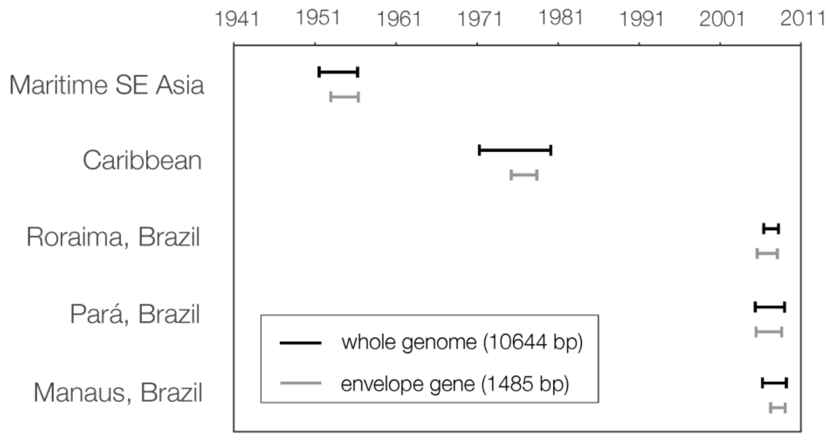

Technical Appendix Figure. Comparison of the divergence time of dengue virus 4 in Maritime Southeast Asia, the Caribbean, and Roraima, Pará, and Manaus States (Brazil) as estimated by using BEAST for full genome and envelope alignments.
